# Controlling Antibiotic Release from Polymethylmethacrylate Bone Cement

**DOI:** 10.3390/biomedicines9010026

**Published:** 2021-01-01

**Authors:** Victoria Wall, Thi-Hiep Nguyen, Nghi Nguyen, Phong A. Tran

**Affiliations:** 1 Faculty of Medicine (Princess Alexandra Hospital), St Lucia Campus, The University of Queensland, Brisbane, QLD 4072, Australia; Victoria.wall@uq.net.au; 2 Interface Science and Materials Engineering Group, School of Mechanical, Medical and Process Engineering, Queensland University of Technology (QUT), 2 George Street, Brisbane, QLD 4000, Australia; 3 Tissue Engineering and Regenerative Medicine Department, School of Biomedical Engineering, International University, Ho Chi Minh City 70000, Vietnam; nthiep@hcmiu.edu.vn (T.-H.N.); nghinguyen.295@gmail.com (N.N.); 4 Vietnam National University, Ho Chi Minh City 70000, Vietnam; 5 Centre for Biomedical Technologies, Queensland University of Technology (QUT), 2 George Street, Brisbane, QLD 4000, Australia

**Keywords:** antibiotics, delivery, bone implant, bacterial infection, cement, PMMA, release

## Abstract

Bone cement is used as a mortar for securing bone implants, as bone void fillers or as spacers in orthopaedic surgery. Antibiotic-loaded bone cements (ALBCs) have been used to prevent and treat prosthetic joint infections by providing a high antibiotic concentration around the implanted prosthesis. High antibiotic concentrations are, on the other hand, often associated with tissue toxicity. Controlling antibiotic release from ALBCS is key to achieving effective infection control and promoting prosthesis integration with the surrounding bone tissue. However, current ALBCs still need significant improvement in regulating antibiotic release. In this review, we first provide a brief introduction to prosthetic joint infections, and the background concepts of therapeutic efficacy and toxicity in antibiotics. We then review the current state of ALBCs and their release characteristics before focusing on the research and development in controlling the antibiotic release and osteo-conductivity/inductivity. We then conclude by a discussion on the need for better in vitro experiment designs such that the release results can be extrapolated to predict better the local antibiotic concentrations in vivo.

## 1. Prosthetic Joint Infections and Local Antimicrobial Delivery Strategy

Prosthetic joint infection (PJI) is the most common cause of total knee arthroplasty (TKA) failure and the third most common cause of total hip arthroplasty (THA) revision [1,2]. PJIs are caused by surgical site seeding of bacteria during surgery or systemic spreading of bacteria from other sites [3]. Acute postoperative PJIs (i.e., occurring within 3 months post-surgery) are usually due to opportunistic virulent organisms such as *Staphylococcus* species, β-haemolytic streptococci and aerobic Gram-negative organisms, while delayed and chronic infections are caused by less virulent organisms such as coagulase-negative staphylococci and *Cutibacterium* species [4,5,6]. PJIs occur at a rate of between 0.2% to 2% yet they are a big problem considering the fact that in the US alone, about 3.5 million total knee arthroplasties are performed every year by 2030 and the cost of revision surgeries runs as high as $50,000 per patient [1,7].

PJIs present a specific challenge due to the formation of protective biofilms on the foreign biomaterials (i.e., implants, cements) or on the surrounding tissue [8]. Bacteria proliferate and are protected in the biofilm from the host immune system or antibiotic treatment [9]. The penetration and diffusion of systemic antibiotics is severely affected due to the biofilm’s extracellular polymeric substance (EPS), local devascularization and fibrous scar formation [10]. The decreased ability of systemic antibiotics to penetrate the biofilm usually necessitates further surgery in order to successfully manage infections [3].

Currently, PJIs are treated by extensive debridement with or without removal of the prosthesis [3]. Debridement, antibiotics, and implant retention (DAIR) is often for acute infections (occurring within one-month post implantation) and can achieve success rates of 80% [11]. DAIR can only be used in patients where the implant remains well-fixed [3]. When prosthesis exchange is necessary, a new implant is inserted (in one-stage procedures) or antibiotic loaded spacers are placed into the wound to clear the infection before insertion of a new implant (in two-stage procedures) [12].

Two-stage arthroplasty with implant exchange has the highest reported success rate (greater than 87%) and is considered the gold standard corrective procedure for infected devices [3]. In this procedure, compromised soft tissue and bone are first debrided, followed by the complete removal of implant material, followed by implantation of an antibiotic-impregnated spacer and six weeks of concurrent systemic antibiotic therapy [13]. Reimplantation following an infected implant carries a much higher risk of infection than the original surgery and the increased likelihood of repeat surgeries or life-long oral antibiotic treatment to prevent infection recurrence [9,14]. 

Systemic prophylactic antibiotics have been the well-established clinical practice to avoid serious consequences of postoperative surgical site or implant infection. Prophylactic antibiotic use is often indicated for up to 24 h before prosthetic large joint replacements, internal fixation of large bone fractures, and for procedures involving insertion of prosthetic or allograft material [15]. Antibiotic guidelines vary between regions and clinicians are guided by their local guidelines which take local microbial resistance and sensitivity into account. For example, Australian Therapeutic Guidelines [16] for prophylaxis treatment in orthopaedic surgery involving open fractures recommend intravenous (IV) cephazolin 2 g (child: 30 mg/kg up to 2 g), 60 min (ideally 15–30 mins) prior to surgical incision. For patients infected with methicillin-resistant *Staphylococcus aureus* (MRSA), vancomycin (adult and child) 15 mg/kg IV, 30–120 mins prior to surgical incision at 10 mg/min is added to cephazolin. While incidental bacteraemia can theoretically occur in all surgical procedures, antibiotic prophylaxis is not indicated for routine arthroscopy procedures (i.e., those that do not involve insertion of prosthesis or avascular tissue) even if the patient has a pre-existing joint prosthesis.

While prophylactic systemic antibiotics have been the gold standard for reducing post-surgery infection risks, they have been recognized as significantly less effective in surgeries involved implantation of foreign materials such as a prostheses or bone cements due to bacterial biofilm formation and the compromised local immune response [17]. As a result, local prophylactic antimicrobial delivery has emerged over the last several decades as a better means of infection control for these surgeries. This local delivery mode is designed to rapidly reach high-levels of antibiotic at the surgical site, and have minimal systemic circulation and therefore avoid systemic toxicity. Antibiotics have been locally delivered from implant coatings, bone cements, spacers, polymer beads, hydrogels and can be used in combination with systemic intravenous antibiotics [18,19]. Infection rates are generally reduced across all the different coating technologies when compared to systemic antibiotic only groups [20]. Controlling antibiotic release is key to achieving effective infection control without toxicity to local issues.

In the sections below we focus on antibiotic delivery from polymethylmethacrylate (PMMA) bone cement for the prevention and treatment of bacterial prosthetic joint infections. PMMA is the most commonly used cement and thus presents exciting opportunities for further improvement. First, the key consideration in using local antibiotics is briefly discussed to provide a background for discussions on the current state of antibiotic-loaded PMMA bone cements. We then discuss the major areas of research and development including antibiotic choice, their stability and mechanisms for controlling antibiotic release. We conclude by discussing the future perspectives of antibiotic delivery from PMMA and offer our opinions on developing better ALBCs.

## 2. Antibiotic Therapeutic Efficacy and Tissue Toxicity Consideration

### 2.1. Therapeutic Level, Duration and Effectiveness

The minimum inhibitory concentration (MIC) is among the most important parameters in assessing an antibiotic’s effectiveness. MIC is defined as the lowest concentration of an antimicrobial substance that completely inhibits the growth of the organism in vitro and thus varies depending on the specific strain of bacteria being targeted and the agent [21]. The measured antibiotic MIC can greatly vary according to resistance and susceptibility profiles and subspecies of the bacteria. The MIC for a particular species must be experimentally determined. 

Another important parameter is antibiotic effectiveness (AE), which means the period in which the dosage is above the MIC. As a result, it depends on the means of delivery and can be calculated as the area under the curve (AUC) that is above the MIC level. Efficacy is evaluated by the ratio of AUC to MIC and using antimicrobial agents and delivery routes having a high AUC and low MIC is more desirable. For example, an AUC/MIC ratio of at least 400 was associated with successful eradication in adults with MRSA lower respiratory tract infections [22]. 

### 2.2. Local Tissue Toxicity

High concentrations of antibiotics can be toxic to tissue cells including osteoblasts [23] and therefore could affect bone formation, particularly with ALBCs because of the impaired vascularization [24]. Different antibiotics have different toxicity profiles. For example, the antibiotics that are frequently incorporated into bone cements are vancomycin, tobramycin and gentamicin and these have different toxicity levels toward bone-forming cells, osteoblasts. At high concentrations (>2000 μg/mL) vancomycin and tobramycin are less toxic to osteoblasts compared to gentamicin [24]. Vancomycin causes effects on cell death and osteogenic activity occurs at a high dose of 5000 μg/mL, while tobramycin starts affecting cell replication at >500 μg/mL with significant cell death at 5000 μg/mL [24]. Of these three commonly used antibiotics, gentamicin is the most sensitive with toxic effects beginning at a lower level of 200 μg/mL where replication and alkaline phosphatase activity is significantly decreased [24]. However gentamicin toxicity at these lower concentrations has not been shown in another study as described in Table 1 [23]. 

Table 1 shows an example of the toxicity levels of different antibiotics to primary osteoblasts, osteosarcoma and HeLa cell lines [23]. The toxicity profiles of antibiotics change according to different cell types and experimental methods such as exposure duration.

Another parameter dictating the toxicity of an antibiotic toward tissue cells is the duration of exposure and its intracellular accumulation, particularly when high-dose bone cements are used. For example, repeated treatment of clindamycin and erythromycin on osteoblasts have shown antibiotic accumulation intracellularly and resulted in much lower IC_20_s concentrations compared to those of a single application [23]. Additionally, a case of acute renal failure after the implantation of a high-dose ALBC spacer containing both tobramycin and vancomycin was reported [25]. The patient was likely impacted by significant systemic tobramycin absorption due to unexpected poor antibiotic clearance and prolonged exposure to elevated aminoglycoside levels [25].

## 3. Current State of Antibiotic Loaded PMMA Bone Cement

Polymethylmethacrylate (PMMA) remains the most commonly used bone cement and is the focus of this review. PMMA is widely used in total joint replacement surgeries to secure the acetabular and femoral components [26]. Besides, it can facilitate fracture and tumour surgery as well as newer techniques such as percutaneous vertebroplasty and kyphoplasty [27,28]. Additionally, PMMA cement has been explored as a carrier for antibiotics to prevent or treat bacterial infection with different types of antibiotics incorporated in bone cements (Table 2). Among which, antibiotics such as gentamicin, tobramycin and vancomycin are often chosen to have a broad antibacterial spectrum coverage and their combination offers multiple bacteria killing mechanisms hence, low resistant development risks [9]. Gentamicin and tobramycin, as aminoglycosides, are both effective against Gram-positive and Gram-negative bacteria [29,30]. They are bactericidal by irreversibly binding the 30S subunit of ribosomes, inhibiting protein synthesis in bacteria [31]. Vancomycin, a glycopeptide, is effective against Gram-positive bacteria such as *S. aureus* [29]. It weakens the outer peptidoglycan layer of the cell wall, causing leakage of cellular matter and hence cell death [22]. Other antibiotics such as, but not limited to, moxifloxacin, daptomycin, ertapenem, meropenem and cefotaxime are also manually mixed into PMMA if pathogen sensitivity testing is available for targeted treatment [32]. Antibacterial resistance can also be a problem for PMMA bone cements. After the incorporation of gentamicin in bone cements, accompanied by its widespread use in medical practice, gentamicin-resistant strains of *S. aureus* were reported and the use of ALBCs may partly result in antibiotic resistance among pathogens [33]. Therefore the long-term ALBC exposure should be considered as an emerging threat to increased antibiotic resistance in medicine today.

However, the three main considerations in antibiotic-loaded cements are mechanical properties (mostly compressive strength), antibiotic elution and bone-ingrowth. In light of these main design criteria, we will discuss two major factors, the loading dose and mixing techniques of current ALBCs.

### 3.1. Loading Dose

The maximum antibiotic amount loaded into cements is among the most important factors as it can compromise the cement’s mechanical strength [49]. It is thus often recommended not to include antibiotics above 10–15 wt%. Above this, the cement mechanical properties are significantly affected [50]. Manufacturer recommendations for in-theatre addition of antibiotics are generally 5% (*w*/*w*) (e.g., 2 g antibiotic/40 g bone cement) but the loading dose also depends on whether the ALBC is used for prophylaxis against infection or treatment of active infection [51]. Specifically, bone cement is loaded with lower dose for prophylaxis to prevent adverse mechanical effects on the implant but higher doses are required for infection treatment to ensure sustained therapeutic effect of antibiotics. For instance, prophylactic low dose ranges from 0.5–1 g antibiotic/40 g cement powder, treatment dose 1–2 g/40 g powder and high dose (e.g., PROSTALAC) of 3.6 g Tobr + 1 g Vanc/40 g powder [51]. The variety of manufacturers and antibiotic dosages in FDA approved ALBCs are shown in Table 3 [51].

The mechanical properties of ALBCs seemed not significantly change as the antibiotics elute. For example, Duey et al. [52] showed that the ultimate compressive strength of their PMMA with varying amounts of tobramycin and vancomycin remained above the minimum limit of 70 MPa specified in American Society for Testing and Materials (ASTM) F451 [53] over 7 days of elution. The authors also found that there was a minimal drop of compressive strength between the inclusion of 0.5 g of each antibiotic and 1.0 g of each. Another study of Funk et al. [54] also noted that the compressive strength of all cement composites loaded with vancomycin were higher than the weight-bearing threshold of 70 MPa with no significant difference throughout the duration of the study indicating that the elution of antibiotics does not directly affect the mechanical strength of ALBCs. Research also is being pursued to increase the recommended threshold of loaded antibiotics. For example, DePuy International (UK) and Biomet Merck (Germany) have incorporated gentamicin and 15% of chitosan nanoparticles resulting in a strong antibacterial with no significant reduction in mechanical strength [55].

### 3.2. Mixing Methods and Release Characteristics

Surgeons in operating rooms usually employ manual mixing or vacuum mixing as the main methods to incorporate antibiotics into the powder component of the cement; therefore, antibiotic release is greatly dependent on the mixing techniques [56]. Cements produced by manufacturers usually come with only one type of antibiotic, thus they are sometimes added with another kind of antibiotic by surgeons in operation theatres.

**Figure 1:**
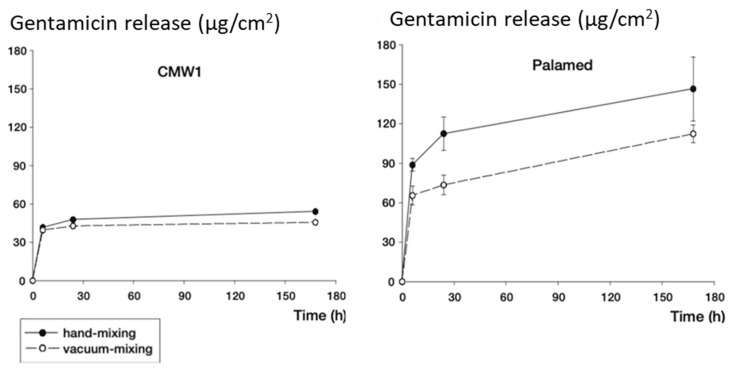
Gentamicin elution from two commercial cements (CMW and Palamed) prepared using hand- or vacuum- mixing technique (adapted and reprinted with permission from Neut et al. [60]).

Manual hand mixing of antibiotics to the cement powder in the operation theatre is commonly used. Hand-mixed cements show higher peak concentrations of antibiotic elution due to increased porosity of the PMMA (5-fold vancomycin, and 2-fold gentamicin elution) when compared to commercially mixed cements (CopalG+V vs. CopalR+G with vancomycin added by hand) [57]. Vacuum mixing is the process of mixing PMMA powder and liquid components under vacuum pressure to reduce the bulk porosity and therefore decreases burst release of antibiotics and improves the mechanics of the cured cement [58]. Compared to hand mixing, Wixson et al. [59], who applied this technology to Simplex P bone cement, a much stronger cement was produced. Neut et al. [60] showed that the release of gentamicin from Depuy CMW and Palamed cements were generally higher with hand-mixed techniques (Figure 1) [61]. However thermal conductivity is elevated due to the removal of voids through vacuum mixing, consequently raising concerns of tissue necrosis [62].

A study on the hand mixing in of a second antibiotic (in this case vancomycin) to a commercially supplied PMMA containing gentamicin found that this addition greatly increased the release rates of both antibiotics [57]. The increased elution from hand mixing in additional antibiotics is possibly due to increased porosity (as a result of air incorporation) and more structural defects within PMMA matrix (as a result of additional antibiotic powder). This study also found that compressive strength of the bone cement was reduced [57].

There are conflicting reports in the literature about the relationship of antibiotic release with antibiotic loading or mixing methods. For example, Minelli et al. [63] combined vancomycin and gentamicin in cement and showed that the combination decreases the release of vancomycin, in contrast to some other studies described above. McLaren et al. [61] showed that there was little to no effect on the antibiotic release from the cement (proprietary or hand-mixed cements containing either gentamicin or tobramycin) when prepared by vacuum mixing or hand mixing techniques.

As antibiotic release can easily reach toxicity level within a confined space coupled with reduced vascularization as in bone implants, research has been strongly focused on achieving higher antibiotic efficacy (e.g., high AUC/MIC ratios, combined antibiotics, release rate control) [64] and improving the osteo-conductivity or osteo-inductivity of antibiotic-loaded PMMA cements. In the section below we review the key areas of significant research and clinical interest.

## 4. Controlling Antibiotic Release from PMMA Cement

### 4.1. Antibiotic Combinations and Antibiotic Heat Stability

Although a number of studies focused on single antibiotic additives to bone cement [32,57,63,65], combining antibiotics into PMMA cement has been widely recognized as the most clinically relevant strategy to expand the antimicrobial spectrum [50,51,56,57,63,65,66,67,68,69,70]. For example, gentamicin, vancomycin and tobramycin are mainly incorporated in cement mixtures due to the ability to target a variety of Gram-positive organisms such as Methicillin-sensitive *Staphylococcus aureus* (MSSA), MRSA, *Streptococcus* and Gram-negative bacteria such as *Pseudumonas aeruginosa* [56]. Glycopeptides such as vancomycin are generally used systemically as a prophylaxis treatment or to medicate serious infections by Gram-positive cocci, including MRSA. It is most efficient at inhibiting cell wall synthesis with Gram-positive organisms and has a bactericidal effect [71]. Regardless, glycopeptides traditionally have poor diffusion characteristics, with small zones of inhibition even at high concentrations [65]. Its application for prophylaxis treatment within implants is permitted by the Therapeutic Goods Administration (TGA) in Australia, however the Food and Drug Administration (FDA) in the USA places restrictions by withholding vancomycin for prophylaxis treatment [51].

Gentamicin, an aminoglycoside effective against both Gram-positive and Gram-negative bacteria, is shown to reduce post-operative infection rates and has previously been included in PMMA for local treatment of infections [31]. The combination of vancomycin and gentamicin is particularly of interest to a number of research groups [63]. As toxicity levels of both therapeutic agents are within the same range, if levels are at least maintained below 700 µg/mL any negative effect on host DNA and cell replication can be avoided [24]. Furthermore, a group demonstrated in vitro that their addition of vancomycin to PMMA had little effects on restricting gentamicin elution, which is beneficial as the action of gentamicin targets streptococci and *P. aeruginosa* [63]. Vancomycin is also a feasible solution to the problem of joint replacements due to its capability of inhibiting Gram-positive cocci, including MRSA. Combined vancomycin and gentamicin also showed synergistic antimicrobial activity [63] against MSSA, MRSA, vancomycin-resistant *S. aureus*, gentamicin-sensitive (GS) and gentamicin-resistant (GR) *S. aureus*, GS and GR *Staphylococcus epidermidis*, and *Escherichia coli*. Yet the indiscriminate use of vancomycin prior to identifying the pathogen is a major criticism in using this combination [56].

The highly exothermic polymerization of PMMA that can result in elevated temperature of 80 °C–90 °C [49] and thus its potential necrotic effects on the surrounding tissue is a key limitation of PMMA [49,72]. ASTM standards F451 specify that acrylic bone cements must not exceed the exothermic temperature of 90 °C to avoid tissue damage [53]. The exothermic reaction of PMMA requires careful consideration of heat stability of an antibiotic when it is incorporated into the cement. For example, vancomycin showed reduction in microbiological activity when incubated in PBS solution for 10 days at 37 °C [65]. Beta-lactam antibiotics are highly fragile and unstable. At 83 °C, gentamicin showed a 25% degradation yet only slight decrease in its activity in a disk diffusion assay [73].

### 4.2. Controlling Antibiotic Release and Osteo-Conductivity/Inductivity

The release of the loaded antibiotics from bone cements is a major design criterion. The release needs to quickly reach a therapeutic level (i.e., MIC) locally and remain above this level for a desirable duration without causing cytotoxic to host cells and tissue. It has been suggested that this time duration is between 4 weeks and 12 weeks depending on the specific application. For example, a spacer would remain in the patient for between 6 and 12 weeks in two-stage hip arthroplasty operations [74] and thus a local antibiotic concentration at or above MIC level during this period is highly desirable.

However, bone cements currently on the market vary in their ability to sustain antibiotic elution with many showing a rapid decline of antibiotic release [10,75] or incomplete release of the incorporated antibiotics due to the hydrophobic nature of the PMMA [76]. Ensing et al. [75] showed release of gentamicin and clindamycin from Copal bone cement (Biomet Merck, Darmstadt, Germany) over a 28 day period, with a prolonged inhibition of GS *S. aureus* and GR coagulase negative *S. aureus* over this period. The PalacosR-G bone cement (Schering-Plough, Maarssen, The Netherlands) however, failed to provide continuous, significant release of gentamicin past the first 24 h [45]. Rate reduction following the initial burst release is a common feature in all antibiotic-loaded cements. This reduction is also dependent on the antibiotics and the mixing method. Antibiotics such as moxifloxacin, daptomycin, ertapenem, meropenem and cefotaxime displayed a slower rate reduction [32]. Rifampin showed the least rate of reduction over time until approximately day 24 when it experienced a sharp decline. Rifampin however was found to interfere with polymerisation of PMMA and negatively affect mechanical properties [32].

**Figure 2:**
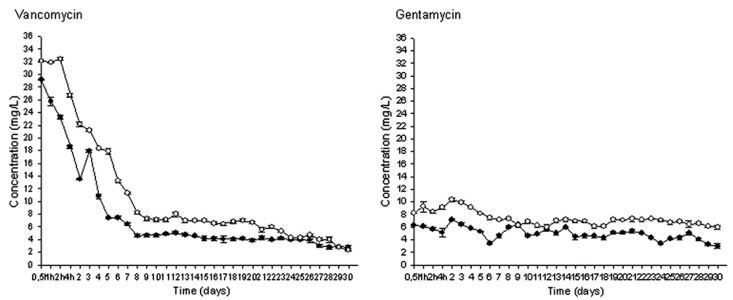
Release of vancomycin and gentamicin (mg/L) over the course of 30 days from PMMA [32]. Filled circles, 10% *w*/*w* concentration. Open circles, 20% *w*/*w* concentration. (Reprinted with permission from Gálvez-López et al. [32]).

The intrinsic different physicochemical properties of antibiotics (such as molecular weight, crystallinity, charges, solubility) have been suggested as a key reason for the difference in their release from cements [77,78]. For example, vancomycin (weak net positive charge, molecular weight (MW) of 1449.3 g/mol) [77] and gentamicin sulphate (strong net positive charge, MW of 516.6 g/mol) [78] have been shown by many groups including ours to release with considerably different kinetics from bone cements. Figure 2 shows vancomycin released from PMMA with an initial burst in the first four hours, followed by a steep decline until day nine, then continuing a constant release before another gradual decrease as it approaches day 30 [32]. Gentamicin on the other hand, displayed a consistent release from the bone cement over the period of 30 days [32].

There is also variation in the elution levels from reports in the literature [79]. Table 4 shows the large variation in the elution profiles among several commonly used methods of custom-mixing ALBCs.

In summary, the inconsistency in elution from ALBCs remains a problem that has not been fully and uniformly addressed. Research to address consistency in this area primarily involves the use of biomaterials as a carrier for the antibiotics or as an additive to bone cements to achieve predictable, extended and complete release of antibiotics. Importantly, these biomaterials are also often chosen to address the poor bone in-growth of PMMA cement. Below we review the two common biomaterials that have been key to this research.

### 4.3. Controlling Antibiotic Release and Improving Bone in-Growth by Biodegradable Polymers—The Case of Poly(lactic-co-glycolic) Acid (PLGA)

Using PLGA to control antibiotic elution from bone cement has been largely inspired by the research in PLGA microspheres or beads containing antibiotics. A co-polymer of poly-lactic acid (PLA) and poly-glycolic acid (PGA), PLGA is among the most studied biomaterials for controlled drug delivery systems. PLGA can be dissolved in chlorinated solvents, acetone or ethyl acetate and be readily processed into various forms and to encapsulate drugs or biomolecules [85].

Perhaps the most important characteristics of PLGA in drug delivery is its biodegradation rate which can be tailored to range from several weeks to months by varying the PLGA molecular weight, PLA to PGA ratio and end-group functionalisation [85]. PLGA undergoes degradation of its ester linkages in an aqueous environment through 4 stages [85]. During the initial stage, water penetrates into the polymer through hydration causing Van der Waal’s forces and hydrogen bonds to be disrupted and polymer swelling. Covalent bonds are then cleaved causing the molecular weight to be reduced. As it enters the third stage of degradation, carboxylic acid end groups catalyse the degradation process and cleaves backbone covalent bonds that promote further mass loss. Smaller fragments are further broken down into molecules that are soluble in the aqueous environment, completing the degradation of PLGA. A higher content of crystalline lactic acid in PLGA (i.e., 85:15) results in a more crystalline, less hydrophilic co-polymer PLGA that absorbs less water and thus has a lower degradation rate compared to PLGA of higher PGA content such as PLGA 50:50 [85].

As a result of these physicochemical properties of PLGA, it has been extensively investigated as a biomaterial for tissue repair, implants, and drug delivery (Table 5) [85].

**Figure 3:**
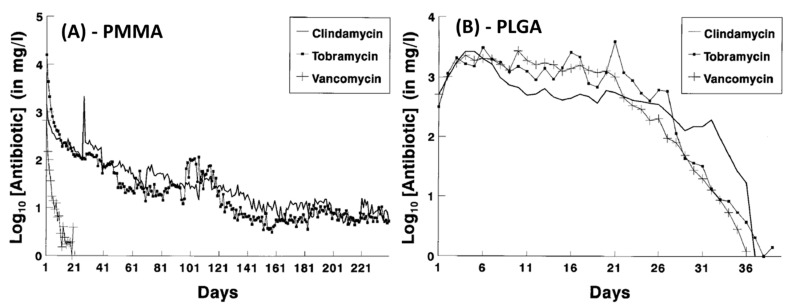
In vitro elution profile of clindamycin, tobramycin and vancomycin from PMMA beads (**A**) and PLGA beads (**B**) (Adapted and reprinted with permission from Mader et al. [86]).

PLGA has been extensively investigated for encapsulating antibiotics and controlling their release. Compared to eluting from PMMA, antibiotic eluting from PLGA can be much more readily controlled thanks to the tailorable crystallinity, swelling and degradation of PLGA. Mader et al. [86] demonstrated that while PMMA beads only deliver adequate doses of vancomycin for 21 days, PLGA beads impregnated with antibiotics showed significant improvement in prolonging this duration to 36 days and considerably modify the release kinetics (Figure 3). The release from PMMA exhibited the usual rapid decrease in elution and elution rates but PLGA beads showed increased release during the first 4–5 days followed by a relatively consistent, zero order-like kinetic to day 20 before it started to decrease sharply [86].

**Figure 4:**
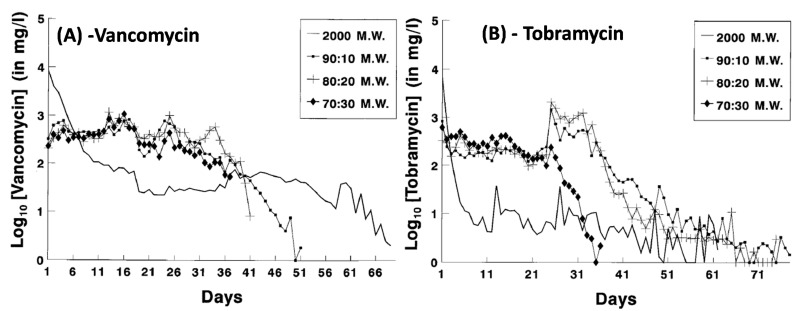
Vancomycin (**A**) and tobramycin (**B**) release from 2000 MW—PLA and PLGA with different lactide:glycolide copolymer ratios. (adapted and reprinted with permission from Mader et al. [86]).

By changing the co-polymer composition, release profiles can also readily be tailored. Figure 4 indicates that vancomycin release sustains a longer and high release until day 25–26 with 70:30, 80:20 and 90:10 PL:CG ratios. A ratio of 90:10 shows the most efficient elution profile, lasting around 50 days before the vancomycin entered complete elution. At the highest molecular weight of PLA (2000-MW), vancomycin and tobramycin were shown to immediately release within the first day of in vitro testing, demonstrating unsatisfactory results due to the burst release. PLGA ratios of 90:10 and 80:20 display a sharp increase in tobramycin release around day 25, while at this same day the 70:30 PLGA indicates a sharp decline in tobramycin release (Figure 4) [86].

### 4.4. Loading Methods

**Figure 5:**
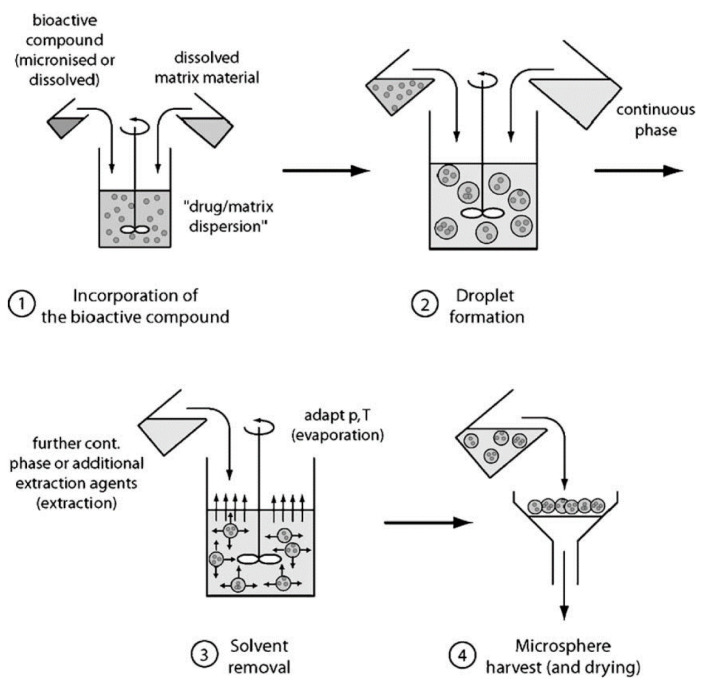
Schematic of solvent extraction/evaporation process to incorporate drugs into PLGA microsphere. (Reprinted with permission from Freitas et al. [87].).

Incorporating antibiotics into PLGA varies in difficulty depending on the desired forms or shapes to be fabricated, such as clumps or microspheres. The encapsulation of bioactive materials into PLGA has been extensively reviewed (e.g., Freitas et al. [87] and Li et al. [88]). The most frequently reported methodology is based on water in oil in water double emulsions (Figure 5) [87].

Incorporating antibiotics into PLGA microsphere and then into PMMA cement is a straightforward process. For example, Spicer et al. [89] used 50:50 PLGA (61 kDa and 37 kDa) to load with antibiotics using this emulsion technique. An internal phase was prepared from 325 mg mL^−1^ colistin dissolved in 0.4 wt% poly(vinyl alcohol) (PVA). Oil phase was 50 mg mL^−1^ PLGA in methylene chloride. The oil phase was then added to the internal phase at a ratio of 20:1 and homogenized. This emulsion was then added to an external phase consisting of 0.4 wt% PVA with 0.5M NaCl at a ratio of 1:10. Colistin impregnated PLGA particles were added at 11 wt% to the powder phase of the bone cement before mixing with the liquid phase according to manufacturer instructions. Colistin release from PLGA incorporated into PMMA bone cement showed an initial burst, followed by a lag phase dependent on the degradation of PLGA, then a second gradual release of antibiotics.

**Figure 6:**
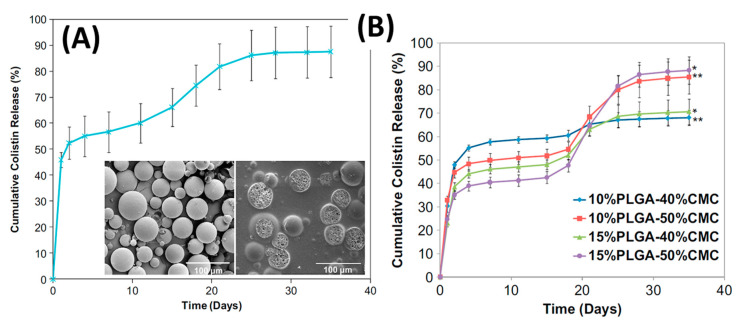
(**A**): Cumulative release of colistin from PLGA microspheres showing initial burst, lag phase and a second slower release. SEM images of external and internal PLGA microsphere morphology. (**B**): In vitro colistin release from PMMA cements modified with CMC and antibiotic-loaded PLGA microspheres showed that higher porosity compositions had greater cumulative release of antibiotic, but it occurs in the second release phase. (Adapted and reprinted with permission from Shi et al. [90]).

Shi et al. [90] modified bone spacers by incorporating 10–15 wt% antibiotic loaded PLGA microspheres and 40–50% carboxymethylcellulose (CMC) hydrogel, to increase porosity, into PMMA bone cement. PLGA microspheres were impregnated with colistin following the method of water in oil in water double emulsion, then mixed with PMMA powder phase. This powder mixture was combined with the hydrogel then the MMA liquid phase was added causing polymerisation and trapping of PLGA microspheres. These highly porous structures allowed for colistin continuous release over 5 weeks at levels well above the MIC (Figure 6A). The cumulative release of colistin is dependent on the composition of the construct it is within with higher porosity compositions (i.e., 50% hydrogel) having a higher second release of antibiotic (Figure 6B). Incorporating particles into PMMA, be those PLGA microspheres, antibiotics, or CMC, results in a trade-off between antibiotic release efficiency and a reduction in PMMA strength [49,50,90].

**Figure 7:**
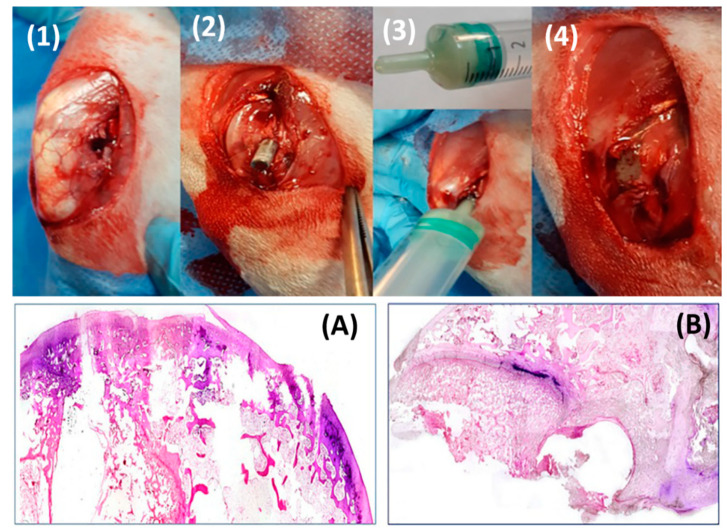
Surgical procedures: (1,2) insertion of the contaminated rod, (3,4) sealing the defect with bone cement. More extensive bone destruction was observed in the antibiotic-containing PMMA control group (**A**) compared to experimental PLGA-modified antibiotic-containing PMMA (**B**) (Adapted and reprinted with permission from Azuara et al. [91]).

Azuara et al. [91] added PLGA into commercial vancomycin or linezolid-containing PMMA cements (Palacos^®^ R) and tested them in a cemented implant model in rabbits. The authors substituted 45% of the solid phase in the commercial cements with PLGA microspheres and used them to secure *S. aureus*-contaminated hydroxyapatite rods (Figure 7). After 3 weeks, the bone destruction caused by bacterial infection was found to be mild in the experimental groups compared to moderate to severe in the cements without PLGA.

### 4.5. Controlling Antibiotic Release and Improving Bone in-Growth by Inorganic Biomaterials—The Case of Calcium Phosphate

The other key biomaterial used to modify PMMA and improve antibiotic elution characteristics is calcium phosphate (CaP) materials. This type of modification is particularly promising because CaP is expected to also encourage bone ingrowth into the bone cement via osteo-conductivity or ions-induced osteo-induction mechanisms [92].

CaP materials such as α-tricalcium phosphate (α-TCP), β-tricalcium phosphate (β-TCP), hydroxyapatite (HA) have been incorporated to PMMA bone cements because of the bioresorbable, osteoconductive or osteoinductive properties of CaP from the release of calcium and phosphate ions [93]. In addition, TCP, α-TCP and β-TCP have been shown to increase setting time and lowering peak temperatures of PMMA [94] making the cement easier for surgeons to handle and shape. The addition of α-TCP beads at approximately 100 µm diameter to the PMMA matrix has also decreased the curing process [95], addressing some concerns about thermal damage to surrounding bone tissue. Lin et al. [55] proposed chitosan/β-TCP composites as an additive to PMMA to enhance cement biocompatibility and reduce its curing temperatures. HA, a bone-mimetic mineral has also been incorporated into PMMA to improve the biological and mechanical properties of the acrylic bone cement, decrease the porosity of cured cement and facilitate heat dissipation [96].

Fini et al. [97] modified PMMA with α-TCP particles (<250 µm in size) and investigated it in vitro and in rabbit bone. Compared to PMMA alone, the α-TCP—modified PMMA significantly increased osteoblast viability, activity and interleukin-6 levels. Higher degradation rate allowed bone growth within and around the TCP bead and led to trabecular and cortical bone integration with the experimental cement. and α-TCP implants had. There was increased colonisation by osteocytes, increased osteoblast activity, osteoinduction, osteoconduction, and bone remodelling due to the synergic effect of the bioactive ceramic TCP and the higher porosity of PMMA.

A key parameter in using CaP materials in PMMA cement is their particle size. As the pore size suitable for bone ingrowth into a porous matrix is between 150–400 µm [55], the resorbable CaP additives to PMMA should be chosen to create these pore sizes upon their resorption to encourage strong bone ingrowth into the PMMA cement. An in vivo rabbit model study showed the use of β-TCP in PMMA (at 30 wt%) to achieve 250 µm bone penetration after 8 weeks while the absence of TCP in bone cement showed no penetration [98].

### 4.6. CaP Materials as Additives to PMMA Bone Cement to Control Antibiotic Release

Giavaresi et al. [99] incorporated β-TCP (24.7 *w*/*w*%) or barium sulfate (23 *w*/*w*%) to a commercial PMMA cement (Mendec Spine) to make spacers. Adding TCP significantly increased the porosity (from about 3% to 21%) and pore connections (from ~0.5% to >90%), which facilitated efficient antibiotic adsorption and release. Antibiotics were loaded into the spacers by simply immersing in low or high concentration solutions of gentamicin (10 mg/mL or 112 mg/mL) in combination with vancomycin (10 mg/mL or 113 mg/mL). The spacers prepared with TCP adsorbed more than 2.5 times as much antibiotics as the spacers with barium sulfate [99].

**Figure 8:**
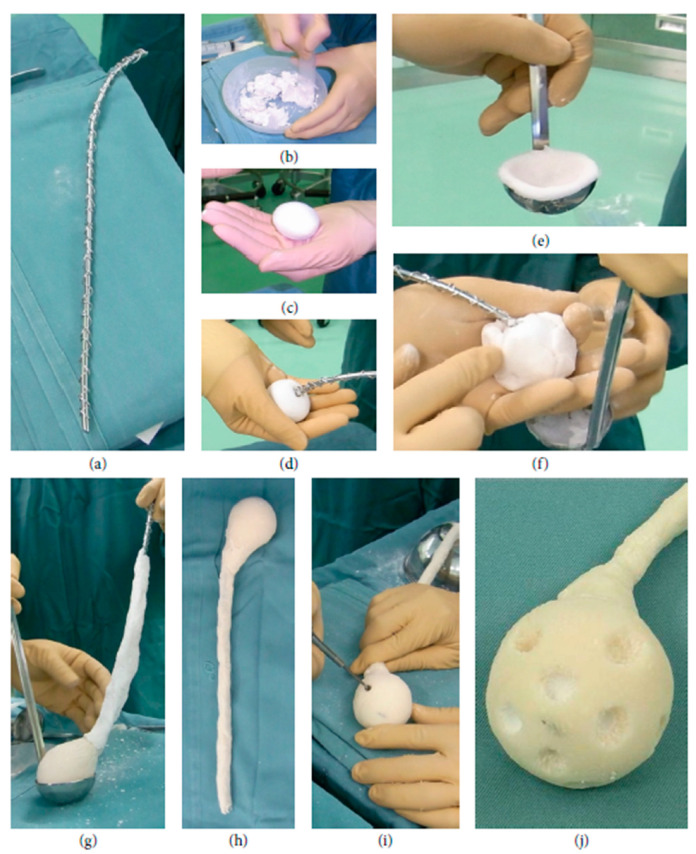
(**a**) An Ender nail was used as a stabilising core to help removal and avoid breakage. (**b**–**d**) The femoral head was constructed from a ball of TCP+Vancomycin paste. PMMA loaded with TCP, gentamicin and vancomycin was used to wrap the rod (**e**–**h**) and the ball to form a complete nail. (**i**,**j**) Holes were drilled through the PMMA to allow efficient delivery of the TCP+antibiotic core. (Reprinted with permission from Uchiyama et al. [100]).

The efficacy of TCP-modified antibiotic-containing PMMA cement was also demonstrated on patients. Uchiyama et al. [100] used a specially constructed bone spacer (Figure 8) made from PMMA bone cement with α-TCP mixed with powdered antibiotics (0.5 g vancomycin, 60 mg gentamicin) in 2-stage revision treatment for infected total hip arthroplasty. The authors showed that there was no repeat infection in 33 out of the 36 hips (success rate 91.7%).

**Figure 9:**
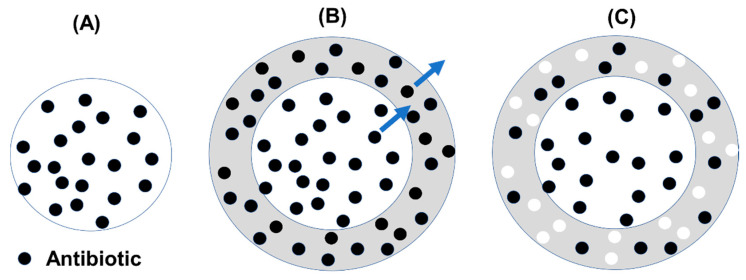
A new design for controlling antibiotic elution from PMMA cements by using coated ceramic particles as additives [102]. Porous ceramic particles are loaded with antibiotics by absorption (**A**). These particles are then coated in a biodegradable polymer coating consisted of PLGA which also contains antibiotics (**B**). The antibiotics in the biodegradable polymer coating release as the polymer swells and degrades, and create voids in the coating (**C**). The antibiotics in the ceramic particles release through these voids and the swollen/degrading polymer. These coated ceramic particles are designed to be mixed into PMMA to form an antibiotic-loaded cement whose antibiotic release can be controlled by multiple factors such as the antibiotic loading, the coating layer, the particle loading of the cement. The ceramic particles can be chosen to degrade, creating large pores in the cement which will facilitate bone ingrowth.

Calcium HA spacers have been compared with PMMA only spacers in a *Staphylococcus*-induced osteomyelitis model in rats [101]. The HA loaded with vancomycin show significantly lower osteomyelitis rates and had the additional benefit of being almost completely resorbed. In addition to using CaP materials as a carrier for antibiotics, they can be further manipulated to increase the level of control over antibiotic elution when incorporated into PMMA cements. Jefferey et al. [102] have developed a new concept of controlled delivery that combines the osteo-conductive/osteo-inductive properties of CaP materials and elution control properties of PLGA. In this design, antibiotics are absorbed into porous CaP microspheres that are then coated with PLGA (Figure 9). As the incorporated antibiotic will need to diffuse out of the CaP spheres, through the PLGA layer before being released from the hardened PMMA cement, it is envisioned that this design would significantly increase the antibiotic effectiveness (measured by the area under the release curve that is above MIC level—AUC). This design also provides multiple mechanisms to further tailor the release profiles to match different clinical needs. Importantly, it is also expected that the gradual degradation of PLGA and the resorption of CaP spheres would create voids in the cements and induce bone ingrowth into these pores.

## 5. Concluding Remarks with an Emphasis on the Current Knowledge Gap

**Figure 10:**
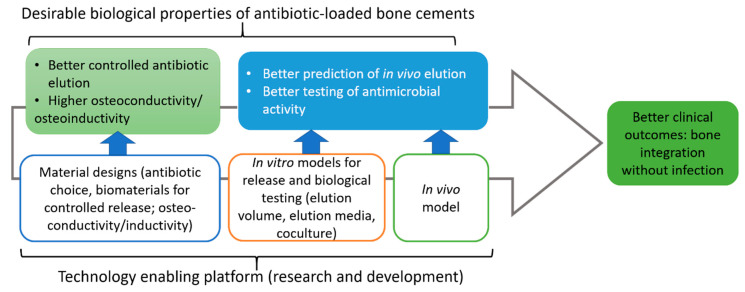
Designing better ALBCs.

Bone cements are an important component in orthopaedic surgery. As bacterial infection remains a significant clinical risk to any foreign materials implanted into the body, bone cements present both challenges and opportunities to address this risk. Incorporating antibiotics into bone cements, particularly PMMA has long been explored to reduce post implantation infection risk or to treat existing infections (hence, lowering the recurrence risk). Current antibiotic-loaded PMMA cements still need significant improvement in terms of the types of antibiotics, their loading dose, mixing methods and their elution profiles as these factors greatly dictate the antimicrobial efficacy, tissue toxicity, bone ingrowth and cement mechanical strength. Better antibiotic-loaded cements require targeted research and development of material designs and in vitro and in vivo testing to deliver cement design criteria that ensure better clinical outcomes of bone integration without infection (Figure 10).

We believe that what has been largely overlooked in the development of ALBCs is the link between in vitro and in vivo testing. Designing ALBCs with more predictable in vivo antimicrobial efficacy, bone formation or bone ingrowth is a non-trivial task because of the complexity of in vivo release. Clearly, antibiotic release needs to be investigated in conditions that simulate the in vivo environment as the release in the body is greatly dependent on the tissue microenvironment [79]. Release profiles could be directly measured in vivo by sampling the environment surrounding bone cement in experiment animals yet their bone volumes are radically different to humans and the experiments would need a large number of animals to provide statistically reliable measurement.

In vitro release experiments, on the other hand, offer good control over reproducibility yet have several crucial limitations that need to be addressed. So far, research groups have mainly tested in vitro antibiotic release from cements in PBS and under sink conditions which gives understanding of the release mechanisms but is nearly impossible to predict the release kinetics in vivo based on these results. Research groups have increased the complexity of the release media to include proteins or amino acids or in the presence of cells. However, the factor that is perhaps most important to predict in vivo concentration at a certain time point is the volume of liquid available for the cement to release its antibiotics. This volume, referred to as ‘*effective release volume*’ is currently very difficult to elucidate. For instance, the volume around a cemented hip stem primarily consists of the gap between the stem and the reamed bone. This gap is initially filled with blood clots which degrade over time. This volume is also connected to the joint space in which the synovial fluid has certain rates of absorption and replenishing [103]. In principle, this volume can be calculated from anatomical data. Our group used this data from existing reports [104,105] and was able to calculate the effective release volume in a cemented hip and knee stem to be approximately 170 mL and 90 mL respectively. This estimation, however, was only possible when we simplified the calculation by assuming negligible fluid resorption/replenishing. Currently, this fluid resorption or replenishing rate is largely unknown. We, however, believe that it can be calculated if there is data from the clinical trials or case reports where both the local concentration and systemic concentration of antibiotics or drugs were measured over time.

## Figures and Tables

**Table 1:** Inhibitory levels of different antibiotics on functions of Primary Human Osteoblasts (PHO), MG63 and HeLa cell lines (adapted and reprinted with permission from Duewelhenke et al. [23]).

Antibiotic	Mean Inhibitory Concentration (μg/mL) for 20% Inhibition (IC_20_) and 50% Inhibition (IC_50_) of Proliferation and Metabolic Activity in Different Cell Types (Highest Concentration Tested Was 400 μg/mL)					
	IC_20_PHO	IC_50_PHO	IC_20_MG63	IC_50_ MG63	IC_20_ HeLa	IC_50_ HeLa
Penicillin G	No effect	No effect	No effect	No effect	No effect	No effect
Flucloxacillin	No effect	No effect	No effect	No effect	No effect	No effect
Amoxicillin	No effect	No effect	No effect	No effect	No effect	No effect
Cefazolin	380, >400	>400, >400	230, 400	>400, >400	270, >400	>400, >400
Vancomycin	No effect	No effect	No effect	No effect	No effect	No effect
Fosfomycin	No effect	No effect	No effect	No effect	No effect	No effect
Gentamicin	No effect	No effect	No effect	No effect	No effect	No effect
Streptomycin	No effect	No effect	No effect	No effect	No effect	No effect
Tobramycin	No effect	No effect	No effect	No effect	No effect	No effect
Ciprofloxacin	70, 260	170, >400	80, 60	160, 150	100, 70	290, 120
Moxifloxacin	80, 190	160, >400	110, 30	230, 170	90, 40	320, 110
Tetracycline	60, ÷	180, ÷	60, ÷	180, ÷	200, ÷	>400, ÷
Rifampin	30, ÷	130, ÷	120, ÷	240, ÷	180, ÷	270, ÷
Clindamycin	40, 340	150, >400	160, 200	250, >400	230, 80	>400, 200
Lincomycin	No effect	No effect	No effect	No effect	No effect	No effect
Erythromycin	30, 210	180, >400	130, 180	300, 310	210, 110	>400, 170
Roxithromycin	20, 110	70, 210	50, 50	180, 110	100, 30	160, 90
Azithromycin	20, 80	25, 160	30, 70	190, 180	110, 50	240, 160
Chloramphenicol	60, 260	230, >400	200, 110	>400, 400	340, 100	>400, 340
Linezolid	240, >400	>400, >400	No effect, 250	No effect, >400	No effect, 300	No effect, >400

÷ ICs not determined; “No effect” means no cytotoxicity was measured after 48 hours of treatment at the maximum 400 µg/mL dose.

**Table 2:** List of antibiotics incorporated in various bone cements and references [33].

Antibiotics	Cement	Reference
Gentamicin/penicillin/erythromycin	Palacos	[34]
Gentamicin	Palacos	[35]
Penicillin/methicillin/erythromycin/lincomycin/nafcillin/polymyxin/colistimate	Simplex	[36]
Gentamicin	CMW/Simplex/Palacos	[37]
Gentamicin/oxacillin/cephazolin	Simplex/Palacos	[38]
Sodium fusidate/gentamicin	Palacos/Simplex/CMW	[39]
Fusidin/clindamycin/gentamicin	Simplex	[40]
Penicillin/gentamicin	Palacos/Simplex	[41]
Gentamicin sulfate/sodium fusidate/diethanolamine	Palacos/CMW	[42]
Ceftriaxone/coumermycin/sulfampicion-methoxozaole/trimethoprim/cephalothin/vacomycin/fusidic acid/ gentamicin/rifampicin/vancomycin	Palacos/CMW	[43]
Vancomycin/amikacin/daptomycin	Palacos/Simplex/Zimmer low viscosity and dough type	[44]
Tobramycin/vancomycin	Palacos/Simplex	[45]
Vancomycin/tobramycin	Palacos	[46]
Tobramycin/vancomycin	Simplex/Palacos	[47]
Vancomycin	Cerafix	[48]

**Table 3:** FDA-approved ALBCs [51].

Product Name	Manufacturers/U.S. Distributors	Cement Type	Dosage of Antibiotic Per 40 g of Bone Cement
Cobalt g-HV	Biomet (Warsaw, IN, USA)	Copolymer high viscosity	0.5 g of gentamicin
Palacos G	Biomet (Warsaw, IN, USA)	Copolymer high viscosity	0.5 g of gentamicin
DePuy 1	DePuy Orthopaedics (Warsaw, IN, USA)	Homopolymer high viscosity	1.0 g of gentamicin
Cemex Genta	Exactech (Gainesville, FL, USA)	Copolymer medium viscosity	0.5 g of gentamicin
VersaBond AB	Smith and Nephew (Memphis, TN, USA)	Copolymer medium viscosity	1.0 g of tobramycin
Simplex P	Stryker Orthopaedics (Mahwah, NJ, USA)	Copolymer medium viscosity	1.0 g of tobramycin
Biomet Refobacin Cement R	Biomet (Warsaw, IN, USA)	High viscosity	2% of gentamicin sulfate
Palacos R+G p (*predecessor Refobacin Palacos*)	Heraeus (Langhorne, PA, USA)	High viscosity	1.96% of gentamicin

**Table 4:** Variation in release from antibiotic-loaded cements. Maximum local antibiotic concentration in hip joint fluid eluted from antibiotic-loaded spacer. Adapted and reprinted with permission from Anagnostakos et al. [79].

Cement Used and Reference	Antibiotic Amount per 40 g Cement Powder	Antibiotic Powder Combination	Hour 1	Day 1	Day 2	Day 7	Week 2	Week 6
			(All Values Are in μg/mL)					
Palacos [80]	2 g	Vancomycin		72		6.6		
	0.5 g	Gentamicin		39		1.9		
Palacos [81]	1 g	Clindamycin						
	1 g	Gentamicin	30.61		53.9			
Cemex [50]	1 g	Vancomycin		28.8				
	0.76 g	Gentamicin		88				
Simplex [82]	4 g	Vancomycin		1538		571.9		>MIC
	4 g	Azertonam		1003		313.6		>MIC
Simplex [67]	3 g	Vancomycin		485.5		76.1		
	480 mg	Gentamicin (liquid)		58.3		14.6		
Prepare in house [83]	2 g	Vancomycin		57				
	-	Gentamicin						
Palacos [84]	2 g	Vancomycin					<MIC	
	0.5 g	Gentamicin					<MIC	
Cemex [68]	150–170 mg	Vancomycin		13.8-40				
	1 g	Gentamicin		15–90				
Palacos [74]	2 g	Vancomycin						50
	1 g	Gentamicin						177
	1 g	Clindamycin						322

**Table 5:** Key physicochemical properties of biodegradable PLGA and its applications (Adapted and reprinted with permission from Gentile et al. [85]).

Polymer	Modulus (GPa)	Elongation (%)	Solvent	Crystallinity (%)	Degradation Time (Weeks)	Applications
Polyglycolide/Polyglactine	7	15–20	Hexafluoroispropanol	45–55	6–12	Suture anchors, meniscus repair, medical devices, drug delivery, orbital floor
Poly(l-lactide)	2.7	-	Benzene, THF, dioxane	37	12–18	Fracture fixation, interference screws, suture anchors, meniscus repair
Poly(d,l-lactide)	-	3–10	Methanol, DMF	Amorphous	11–15	Orthopaedic implants, drug delivery
Poly(d,l-lactide-*co*-glycolide) 85/15	2	3–10	Ethyl acetate, chloroform, acetone, THF	Amorphous	5–6	Interference screws, suture anchors, ACL reconstruction
Poly(d,l-lactide-*co*-glycolide) 75/25	2	3–10	Ethyl acetate, chloroform, acetone, DMF, THF	Amorphous	4–5	Plates, mesh, screws, tack, drug delivery
Poly(d,l-lactide-*co*-glycolide) 50/50	2	3–10	Ethyl acetate, chloroform, acetone, DMF, THF	Amorphous	1–2	Orthopaedic implants, drug delivery
